# Spatiotemporal Distribution and Influencing Factors of Ecosystem Vulnerability on Qinghai-Tibet Plateau

**DOI:** 10.3390/ijerph18126508

**Published:** 2021-06-16

**Authors:** Han Li, Wei Song

**Affiliations:** 1 Key Laboratory of Land Surface Pattern and Simulation, Institute of Geographic Sciences and Natural Resources Research, Chinese Academy of Sciences, Beijing 100101, China; lih.19s@igsnrr.ac.cn; 2 College of Resources and Environment, University of Chinese Academy of Sciences, Beijing 100049, China

**Keywords:** ecosystem vulnerability, spatiotemporal distribution, influencing factors, Qinghai-Tibet Plateau, principal components analysis

## Abstract

As the “Third Pole”, the Qinghai-Tibet Plateau is threatened by environmental changes. Ecosystem vulnerability refers to the sensitivity and resilience of ecosystems to external disturbances. However, there is a lack of relevant studies on the driving factors of ecosystem vulnerability. Therefore, based on spatial principal components analysis and geographic detectors methods, this paper evaluates the ecosystem vulnerability and its driving factors on the Qinghai-Tibet Plateau from the years 2005 to 2015. The results were as follows: (1) The ecosystem vulnerability index (EVI) of the Qinghai-Tibet Plateau is mainly heavy and extreme, showing a gradually increasing trend from southeast to northwest. (2) The spatial heterogeneity of the EVI is significant in the southeast and northwest, but not in the southwest and central parts. (3) Analysis of influencing factors shows that environmental factors have more significant effects on EVI than socioeconomic variables, facilitating the proposal of adequate policy implications. More efforts should be devoted to ecological protection and restoration to prevent grassland degradation and desertification in the high-EVI areas in northwest. The government is also urged to improve the ecological compensation mechanisms and balance ecological protection and residents’ development needs in the southeast.

## 1. Introduction

Ecosystem is the general term for all organisms and environments within a particular space. Ecosystems are complex open systems, mainly including social systems, natural systems and social-natural coupled systems [1]. According to Adger [2], vulnerability is the sensitivity of ecosystem under the stress of natural and social changes due to the lack of adaptability. In recent years, as a result of increased human activities and global climate changes, ecosystems have been under increasing pressure, aggravating their vulnerability towards a series of stressors [3]. Ecosystem vulnerability assessments are therefore critical in global environmental change research [4], providing a decision-making basis and technical support for ecological protection and environmental restoration and governance [5]. Ecosystem vulnerability has become a hot spot of global environmental change and sustainable development research [6,7,8,9]. Understanding the driving mechanisms of regional ecological vulnerability evolution can facilitate the establishment of guidelines for the use and protection of the regional ecological environment [10].

Several studies have considered the impacts of climate change and natural disasters on ecosystem vulnerability. For example, based on the prediction results of temperature and precipitation under low (B1), medium (A1B) and high (A2) emission scenarios, released in the fourth assessment report of the International Panel of Climate Change (IPCC), Gonzalez et al. [11] studied the changes in vegetation vulnerability patterns in global ecosystems in the 21st century. Based on their results, one-tenth to one-half of the global vegetation area may be highly (confidence level 0.80–0.95) to very highly (confidence ≥ 0.95) vulnerable to climatic changes. Similarly, Alexander et al. [12] assessed the vulnerability of tropical ecosystems in southern Ecuador and found differences in ecosystem vulnerability under different climate scenarios. Patrick et al. [13] investigated the vulnerability of 52 major vegetation types in the western United States exposed to changes in temperature and precipitation under RCP 4.5 scenarios (RCP4.5, Representative Concentration Pathway 4.5, a moderate emission scenario proposed by the Coupled Model Intercomparison Project Phase 5). Their results showed that by the middle of the 21st century, 33 vegetation types will be faced with high or very high vulnerability, of which more than 50% will have higher regional vulnerability levels.

In recent years, the vulnerability of different types of ecosystems has gradually been studied, with a higher number of studies on the vulnerability of certain ecosystem systems, such as mining areas, economically developed areas and oceans. For example, Sarah et al. [14] assessed the vulnerability of marine ecosystems in California and found that tidal flats, beaches, salt marshes and intertidal rocky ecosystems were most vulnerable to human activities. Similarly, Zhang et al. [15] investigated the effects of extreme rainfall on ecosystem vulnerability in the middle and lower reaches of the Yangtze River in China and showed that both human-dominated ecosystems (e.g., agro-ecosystems) and natural ecosystems are vulnerable to extreme climate events. The current vulnerability studies of typical ecosystems that are particularly sensitive to global climate change mainly focus on coastal zones [16,17,18] and wetland regions [19,20]. However, there are no studies on the vulnerability of high-elevation ecosystems, such as the Qinghai-Tibet Plateau, the largest and highest plateau in the world. Due to its unique environment, it is highly sensitive to climate change and human activities, with a fragile ecosystem [21].

China is one of the countries with the most vulnerable ecosystem types in the world, and the research on its ecosystem vulnerability began in the 1980s. For example, Niu [22] conducted a study from the perspective of the ecotone. Early studies mainly focused on the impacts of climate change, extreme weather and natural disasters on ecosystems, such as the analysis of the vulnerability of China’s forest ecosystems under global climate change [23]. In the 1990s, socio-economic factors started to become increasingly considered in the assessment of ecosystem vulnerability, such as the relationship between the fragile zone of the ecosystem and the population [24] or the relationship between ecosystem vulnerability and agricultural development [25]. Since the 21st century, natural and socio-economic factors have been regarded as important factors that play a crucial role assessing ecosystem vulnerability, and numerous related studies have been conducted in typical regions, such as the Three Rivers Source [26]. In addition, the vulnerability of different components of an ecosystem, such as grassland ecosystems [27], was further studied.

In recent years, as the government has started to increasingly consider the importance of environmental integrity, substantial investments have been made in the field of ecological protection. For example, in 2020, the Chinese government put forward the concept of building a “beautiful China” and promoted the construction of an ecological civilization. As a consequence, researchers are paying more attention to the evaluation of ecosystem vulnerability in typical regions with serious ecological and environmental problems. Even though in some areas of the Qinghai-Tibet Plateau, studies on ecosystem vulnerability have been performed, there is a lack of consideration of anthropogenic factors [28]. Due to the construction of infrastructure, such as the Qinghai-Tibet Railway, and the development of tourism, the intensity of human activities in the Qinghai-Tibet Plateau has increased sharply. Against the background of the implementation of China’s ecological protection policy, it is now necessary to gain insights into the overall ecosystem vulnerability and driving forces of the Qinghai-Tibet Plateau. Such studies can provide theoretical references for the sustainable development of the Qinghai-Tibet Plateau and put forward feasible suggestions for the protection of this area.

The objective of this study is to explore the temporal and spatial changes of ecosystem vulnerability and the impacts of natural and socio-economic factors on the Qinghai Tibet Plateau. Specifically, we tested two main hypotheses: (1) the spatial distribution of ecosystem vulnerability has significant spatial patterns; (2) the impacts of natural factors on ecosystem vulnerability are greater than those of socio-economic factors.

## 2. Literature Review

### 2.1. The Concept of Ecosystem Vulnerability

Since the concept of ecological vulnerability has evolved from vulnerability, we start with a brief review of the development of the vulnerability concept. There are numerous statements about the concept of vulnerability. In 1945, White et al. [29] put forward the “adaptation and adjustment view” for the first time when studying flood disasters, which marked the beginning of vulnerability research. After that, White [30] defined vulnerability as a system, subsystem or system component due to its exposure and sensitivity, making it susceptible to external disturbance and pressure. Timmerman [31] defined vulnerability as the degree to which a system is adversely affected or damaged. After that, Dow [32], Cutter [33] and the IPCC [34] defined ecosystem vulnerability from different perspectives. In the 21st century, the concept of vulnerability has been widely used in many fields, including sustainable development [35], climate change [3] and ecology [12].

Ecosystem vulnerability was initially introduced into ecology by Clements, with the concept of the “ecological transition zone” [36], and a unified definition of ecosystem vulnerability as not yet been provided (Table 1). At present, the IPCC’s definition of vulnerability has been widely accepted and adopted in the field of climate change research. Based on relevant literature, ecosystem vulnerability can be summarized as the sensitivity and resilience of ecosystems in response to external interference including human disturbance, climate change, etc.

### 2.2. Assessment of Ecosystem Vulnerability

Ecosystem vulnerability studies rely on building assessment models. At present, there is no unified model for ecosystem vulnerability assessment; the common evaluation models include the Pressure-State-Response model (PSR) [21] and the Exposure-Sensitive-Adaptive model (ESA) [40]. Based on the PSR model framework, some scholars have developed a series of models by adding factors, such as the Driving force-Pressure-State-Impact-Response (DPSIR) [41] and the Pressure-Support-State-Response (PSSR) [42]. Similar to the ESA models, there is the Vulnerability-scoping-Diagram (VSD) model [43]. Based on PSR and ESA models, some scholars have also proposed Pressure-Sensitivity-Elasticity (PSE) [44] and Sensitivity-Resilience-Pressure (SRP) models [45]. The ecosystem vulnerability assessment model is developing in the direction of integrating multiple systems and multiple factors.

On the basis of the indicator system, ecological vulnerability assessment needs to be carried out in conjunction with the assessment methodology, such as the hierarchical analysis method [46], the fuzzy evaluation method [47], the artificial neural network method [48], the entropy weight analysis method [49] and the expert scoring method [50]. With the development and application of RS (Remote Sensing), GIS (Geographic Information System), GPS (Global Positioning System) and other technologies, vulnerability assessment results have become more dynamic. For example, Yaw et al. [51] used GIS and RS to analyze the vulnerability of the Niger River Basin and its influencing factors. The spatial principal components analysis method (SPCA), based on principal components analysis and spatial feature extraction, has advantages in ecosystem vulnerability assessment [52]. For example, it not only adds spatial constraints to the traditional PCA but also considers the spatial dependence in data sets.

Since the purposes and regional characteristics of the studies, along with their emphasize, can largely differ, there is no unified index system. In recent years, ecosystem vulnerability assessment indicators for different regions have been selected (Table 2). In this study, the Sensitivity-Resilience-Pressure (SRP) model was used to construct the index system. This model is constructed based on the connotation of ecosystem stability and has been widely used in the Karst Mountains [53], the Yimeng Mountain area [45] and the Shiyang River region [54], among others. Here, sensitivity reveals the resistance of the ecosystem to various disturbances and is usually expressed by topographical and meteorological factors. In contrast, restoration refers to the ability of an ecosystem to be restored to the original state after damage by internal and external interference factors; it is mainly characterized by vegetation factors. Pressure refers to the variety of pressures from anthropogenic interference, often expressed by population pressure and intensity of economic activities. Since ecosystem vulnerability is generally the result of a combination of natural and human activities, the driving factors that affect changes in ecological vulnerability can be divided into two categories: natural and socio-economic factors [10,55].

## 3. Study Area and Data Sources

### 3.1. Study Area

**Figure 1:**
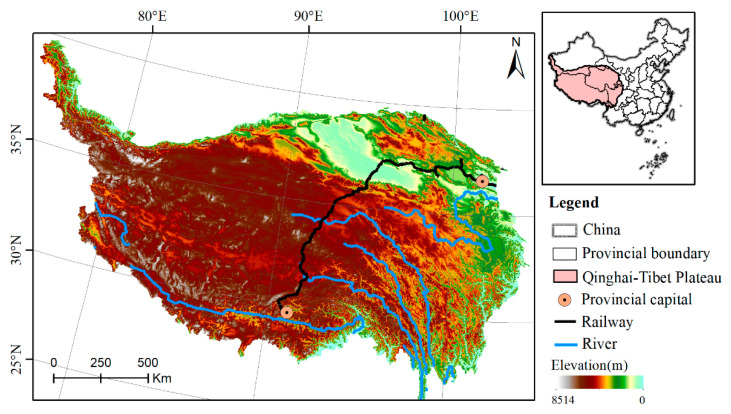
Geographical location of the Qinghai-Tibet Plateau.

The Qinghai-Tibet Plateau in southwest China is the highest plateau in the world, also known as the “Third Pole” (Figure 1). Its average elevation is more than 4000 m above sea level. The administrative regions include Tibet Autonomous Region, Qinghai Province and parts of Xinjiang Uygur Autonomous Region, Gansu, Sichuan and Yunnan Province. It is the birthplace of the Yangtze River, the Yellow River and the Lancang River, among others. The terrain is diverse, containing valleys and basins and the climate is highly complex and largely affected by the terrain. The spatial and temporal distribution patterns of air and heat on the Qinghai-Tibet Plateau are significant. The southeastern area is warm and humid, whereas the northwestern area is dry and cold. The annual average temperature of the entire region ranges between 5.6 and 17.6 °C. Annual precipitation is unevenly distributed, gradually decreasing from 2000 mm to less than 50 mm from southeast to northwest. Under the influence of temperature and precipitation, the surface cover type changes from southeast to northwest, gradually transitioning from forest and shrub areas to grassland, meadow and desert. As a result of overgrazing, the alpine grassland on the Qinghai-Tibet Plateau is subjected to serious desertification. The major ecological issues faced include freeze-thaw erosion, hydraulic erosion, desertification, salinization and water scarcity [58].

### 3.2. Data Sources

For this study, the data used include socio-economic, remote sensing, topographic, meteorological and land use (Table 3) from 2010, 2010 and 2015. They were mostly obtained from the Resource and Environmental Science Data Center of the Chinese Academy of Sciences (RESDC) and include socio-economic (population and GDP (gross domestic product)), topographic (DEM (digital elevation model)), meteorological (annual precipitation and average annual temperature) and land use data with a spatial resolution of 1 km. Remote sensing data were obtained from MODIS (Moderate resolution imaging spectroradiometer) and include NDVI (Normalized difference vegetation index), NP (Net Primary Productivity) and ET (Evapotranspiration). The spatial resolution of NDVI and NP is 1 km ant that of ET 500 m.

All data were preprocessing using the ARCGIS 10.2 software. First, all data were projected into the same coordinate system (WGS_1984_UTM_45N) and then cut into the same spatial boundary according to the study area. Finally, the spatial resolution of data was unified to 1 km by bilinear interpolation. The NDVI represents the monthly data with 12 periods per year, and the annual NDVI was generated by selecting the annual maximum.

## 4. Research Method

### 4.1. Technical Route

**Figure 2:**
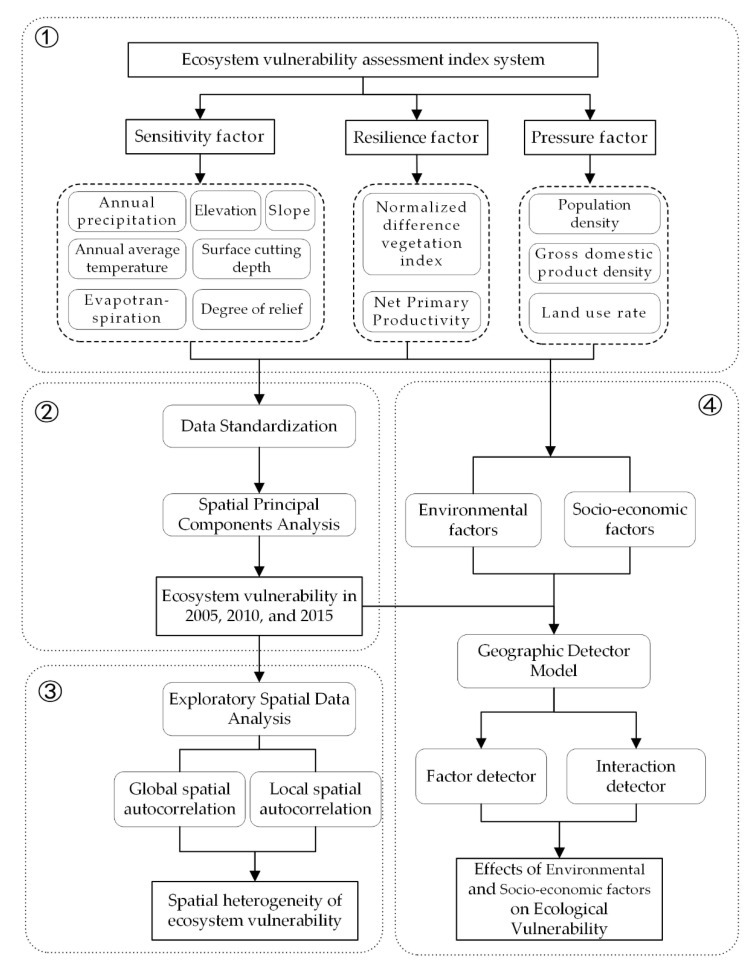
Flowchart showing the process followed in this analysis for assessing ecosystem vulnerability of the Qinghai-Tibet Plateau.

The study was divided into the following four steps (Figure 2):

Step 1: Establishing the ecosystem vulnerability assessment index system. According to the Sensitivity-Resilience-Pressure (SRP) model, the indicators were selected from three aspects: ecological sensitivity, resilience and pressure.

Step 2: Mapping the distribution of ecosystem vulnerability. First, the indicators were standardized and uniformly mapped to the same value range to solve the problem of inconsistent original data units. Subsequently, the spatial scale of ecological vulnerability was determined using the ARCGIS 10.2 software and the SPCA method.

Step 3: Spatial heterogeneity analysis. The spatial and temporal distribution characteristics of ecosystem vulnerability were analyzed via exploratory spatial data analysis.

Step 4: Driving force analysis. Using the factor and interaction detector in the geodetector model, the effects of natural and socio-economic factors on ecosystem vulnerability were analyzed.

### 4.2. Establishing an Ecosystem Vulnerability Assessment Indicator System

In this study, the Sensitivity-Resilience-Pressure (SRP) model was used to construct the index system. The selected 12 indicators were divided into three categories, namely sensitivity, resilience and pressure (Table 4). In previous studies of ecosystem vulnerability in Shiyang River Basin [54], Karst [53] and Yimeng [45] mountainous areas using SRP model, sensitivity is considered to be the product of the interaction between the topographic factors and the distribution of meteorological factors. In this study, annual average temperature, annual precipitation and ET were just selected to reflect the hydrothermal conditions of the ecosystem [10,28]. Here they are not affected as external hazards. Elevation, slope, surface cutting depth and degree of relief were used to characterize the regional topography [61]. Resilience is usually characterized by vegetation factors [62], and NDVI and NPP were selected to reflect vegetation growth. The NDVI can detect the vegetation growth status and accurately reflect the surface vegetation coverage [63]. The NPP not only reflects the productive capacity of vegetation communities, but also represents ecosystem quality [64]. Pressure factors include population density, gross domestic product density and land use rate. Population density and GDP density represent the degree of population and economic concentration, reflecting the interference intensity of human activities. When the disturbance intensity exceeds the carrying capacity of the ecosystem, the ecological environment will be degraded, resulting in increased ecosystem vulnerability [26]. The land use rate (proportion of cultivated land) was selected to reflect the influence of human activities on land use.

### 4.3. Mapping Ecosystem Vulnerability

#### 4.3.1. Data Standardization

Standardization is generally carried out to solve the issue of inconsistent original data units [53]. There are two relationships between ecosystem vulnerability and evaluation factors [65]. The lower the index value, the lower the ecosystem vulnerability, representing a positive correlation. Conversely, there is a negative correlation, that is, the lower the index value, the stronger the ecosystem vulnerability. The maximum-difference normalization method was used to standardize the positive and negative indicators. For the positive indicators in the ecosystem vulnerability assessment index system, the standardized methods are as follows [10]:

(1) $$M_{i}=\frac{X_{i}-X_{min}}{X_{max}-X_{min}},$$

The negative indicators are treated as follows:

(2) $$M_{i}=\frac{X_{max}-X_{i}}{X_{max}-X_{min}},$$

where “*M_i_*” is the standardized value of index i; “*X_i_*” is the initial value of index *i*; “*X_min_*” is the minimum value of index *i*; “*X_max_*” is the maximum value of index *i*.

#### 4.3.2. Spatial Principal Components Analysis

Spatial principal components analysis (SPCA) is a statistical analysis method that converts initial multiple indicators into irrelevant comprehensive indicators by dimension reduction [66,67]. At the same time, the correlation between the original evaluation indexes is reduced, and the information reflected by the original variables is kept to the maximum extent with less comprehensive indices to avoid the repetition of the indicators affecting the accuracy of the evaluation. In this study, we analyzed 12 standardized indices by principal components analysis to generate a new comprehensive index. By solving the correlation coefficient matrix of the index, the feature vector was obtained, and 12 principal component results are acquired. The principal component with a cumulative contribution rate of more than 85% was selected to replace the original index, and the principal factor was determined [67]. On this basis, the comprehensive index of the principal component was calculated as follows [66]:

(3) $$PC_{i}=a_{1i}X_{1}+a_{2i}X_{2}+a_{3i}X_{3}+\ldots a_{ni}X_{n},$$

where “*PC_i_*” is the *i*-th principal component; “$a_{1i}$, $a_{2i}$…$a_{ni}$” are the feature vectors corresponding to the respective index factors of the i-th principal component; “$X_{1}$, $X_{2}$…$X_{n}$” are the respective index factors.

The ecosystem vulnerability index (EVI) was calculated based on the principal components analysis, using the following equation [68]:

(4) $$\mathrm{EVI}=b_{1}PC_{1}+b_{2}PC_{2}+b_{3}PC_{3}+\ldots b_{n}PC_{n},$$

where “EVI” is the ecosystem vulnerability index; “*b*i” is the contribution rate corresponding to the *i*-th principal component; “*PC_i_*” is the *i*-th principal component; “*n*” is the first n principal component whose cumulative contribution rate exceeds 85%. The SPCA in this study was calculated by the ArcGIS 10.2 software. The SPCA results for the years 2005, 2010 and 2015 are shown in Table 5.

To compare the EVI results of several years, the EVI was standardized as follows:

(5) $$K_{i}=\frac{EVI_{i}-EVI_{min}}{EVI_{max}-EVI_{min}},$$

where “*K_i_*” is the standardized value of ecosystem vulnerability in the *i*-th year, with a value range of 0–1; “*EVI_i_*” is the actual value of the ecosystem vulnerability index in the *i*-th year; “*EVI_max_*” is the maximum value of the *i*-th ecosystem vulnerability index; “*EVI_min_*” is the minimum value of the *i*-th ecosystem vulnerability index.

#### 4.3.3. EVI Classification

We used natural breakage classification (NBC) to classify the EVI to reflect different degrees of ecosystem vulnerability. This method is generally used to analyze the statistical distribution of attribute, maximizing the difference between classes [56]. In this study, according to the results of the NBC for 2005, the EVI was divided into five grades, namely, slight, light, medium, heavy and extreme vulnerability (Table 6). Subsequently, the ArcGIS 10.2 software was used to visualize the spatial distribution of EVI.

### 4.4. Spatial Heterogeneity Analysis

The exploratory spatial data analysis method (ESDA) can be used to reveal the spatial interaction mechanism by describing and visualizing the spatial distribution pattern [69]. According to the different scales of analysis, global and local spatial autocorrelation are often used to study the spatial feature of the observation [56]. Here, this was performed using the OpenGeoda 1.16.0.16 software at a spatial resolution of 1 km. Global spatial autocorrelation analysis is mainly used to reflect the cluster degree of similar attributes in a study area [70]. The degree of spatial autocorrelation is usually measured by the Global Moran’s I proposed by Moran [71]. Local spatial autocorrelation is mainly used to measure the spatial correlation and difference between the region of the research target and its surrounding areas [72].

The global Moran index is calculated as follows:

(6) $$I_{i}=\frac{N}{\sum_{i=1}^{N}{\left(x_{i}-\bar{x}\right)}^{2}}\times \frac{\sum_{i=1}^{N}\sum_{j=1}^{N}\left(x_{i}-\bar{x}\right)\left(x_{j}-\bar{x}\right)}{\sum_{i=1}^{N}\sum_{j=1}^{N}w_{ij}},$$

where “*I*” is the Moran index, “*N*” is the number of research objects, “*x_i_*” and “*x_j_*” are the spatial attribute values of the research objects, and “*w_ij_*” is the spatial weight matrix. The value range of “*I*” is [–1, 1]. If the index is greater than 0, the space is positively correlated; if it is smaller than 1, it is negatively correlated. At a value equal to 0, there is no correlation.

The specific equation to calculate local spatial autocorrelation is as follows:

(7) $$I_{i}=\frac{N}{\sum_{i=1}^{N}\left(x_{i}-\bar{x}\right)}\times \left(x_{i}-\bar{x}\right)\times \sum_{j=1}^{N}w_{ij}(x_{i}-\bar{x}),$$

where when *I_i_* > 0, the local space of the research target is positively correlated, and the surrounding area presents a similar attribute value cluster. When the attribute values of the research target area and the surrounding research area are both high, they are hotspot clusters, generally represented by high-high (HH); when the attribute values of the research target area and its surrounding research area are low, they are coldspot clusters, generally represented by low-low (LL). When *I_i_* < 0, the research target’s local space is negatively correlated, and the surrounding area of the research target shows the opposite phenomenon of attribute value cluster. When the attribute value of the research target area itself is high, but that of the surrounding area is low, it is a high-low cluster, generally represented by high-low (HL). When the attribute value of the research target area itself is low, but that of the surrounding area is high, it is a low-high cluster, generally represented by low-high (LH).

### 4.5. Driving Force Analysis

The Geographic Detector Model (GDM) is a set of statistical methods to identify spatial differentiation among the geographical elements. This method can quantitatively analyze the driving mechanisms of geographical phenomena and is widely used to determine the explanatory power of driving factors and the interaction between factors without too many hypothetical conditions [73,74,75].

The GDM includes four detectors, namely risk detector, factor detector, ecological detector and interaction detector. In this study, factor detector and interaction detector were used to analyze the driving factors of ecosystem vulnerability on the Qinghai-Tibet Plateau, with the aim to explore the main driving mechanism of ecosystem vulnerability and to compare the spatial consistency between EVI and evaluation indices. If a factor dominates the cause of vulnerability, vulnerability will exhibit a spatial distribution similar to the evaluation index and the intra-layer variance is lower than the inter-layer variance. Using *q*-statistics to measure the decisive effect of each evaluation index on EVI, the calculation method is as follows [76]:

(8) $$q=1-\frac{\sum_{h=1}^{L}N_{h}\delta_{h}^{2}}{N\delta^{2}},$$

where “*q*” is the explanatory power of the influencing factors to the vulnerability of the ecosystem, “*N*” is the sample size, “*L*” is the classification number of the index factors and “*N_h_*” and $“\delta_{h}^{2}$” represent the variance of h-layer sample size and ecosystem vulnerability, respectively. The value of the *q*-statistic is in the range of [0, 1]; the larger the value, the stronger the explanatory power of the influence factor to the ecosystem vulnerability, and its spatial distribution is consistent with the EVI. When the *q*-statistic is equal to 0, there is no significant relationship between the given influence factor and the EVI distribution. When the value is 1, the impact factor can fully explain the spatial variation of the EVI.

The interaction detector was adopted to reveal the factor explanatory power to the results after multi-factor interaction, that is, whether the interaction of impact factors X1 and X2 will strengthen or weaken the impact on ecosystem vulnerability. The main types are shown in Table 7.

## 5. Results

### 5.1. Spatiotemporal Variations in Ecosystem Vulnerability

#### 5.1.1. Temporal Variations in Ecosystem Vulnerability

**Figure 3:**
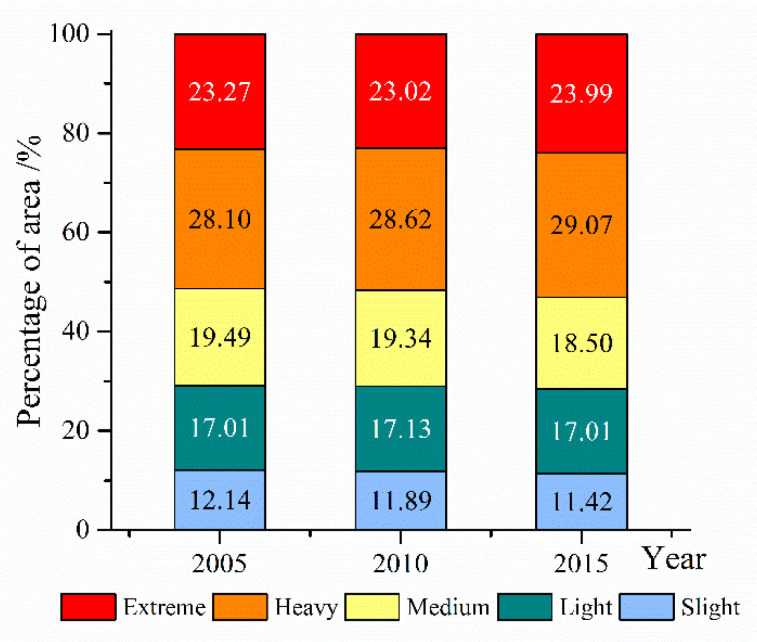
Area proportions of different ecosystem vulnerability levels on the Qinghai-Tibet Plateau in 2005, 2010 and 2015.

The ecosystem vulnerability levels in most areas of the Qinghai-Tibet Plateau were dominated by heavy and extreme vulnerability (Figure 3). In 2005, 2010 and 2015, heavily and extremely vulnerable areas accounted for 51.37, 51.64 and 53.08% of the total area, respectively. Heavily vulnerable areas accounted for the largest proportions, namely 28.10, 28.62 and 29.07%, respectively. From 2005 to 2015, the proportions of slightly and medium vulnerable areas decreased by 0.72 and 0.99%, respectively. The proportion of slightly vulnerable areas did not change, whereas those of heavily and extremely vulnerable areas increased by 0.97 and 0.77%, respectively.

**Figure 4:**
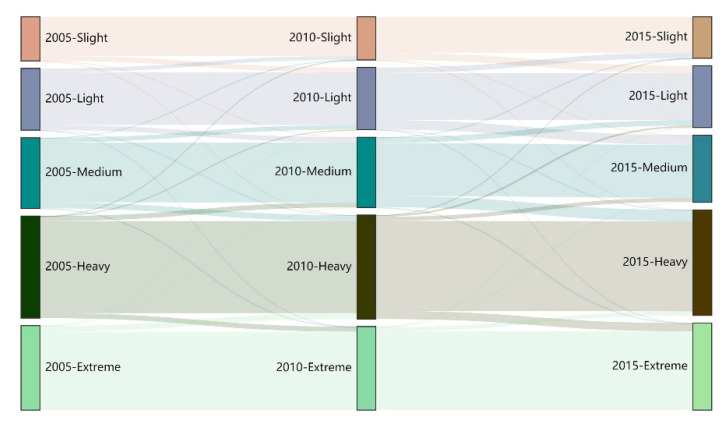
Area conversion of ecosystem vulnerability grades on the Qinghai-Tibet Plateau in 2005, 2010 and 2015.

The transition areas of ecosystem vulnerability level were calculated for 2005, 2010 and 2015 (Figure 4). Area conversion mainly occurred between adjacent levels. For example, the increasing areas of heavily and extremely vulnerable areas were former medium and heavily vulnerable areas. From 2005 to 2015, highly vulnerable areas were mainly a result of the transformation of medium and extremely vulnerable areas, accounting for 76.32 and 23.64%, respectively. The extremely vulnerable areas are almost entirely transformed into heavily vulnerable ones. The main types of ecosystem vulnerability scale conversion include medium to high, light to medium, slight to light and high to extreme vulnerability.

#### 5.1.2. Spatial Variations in Ecosystem Vulnerability

**Figure 5:**
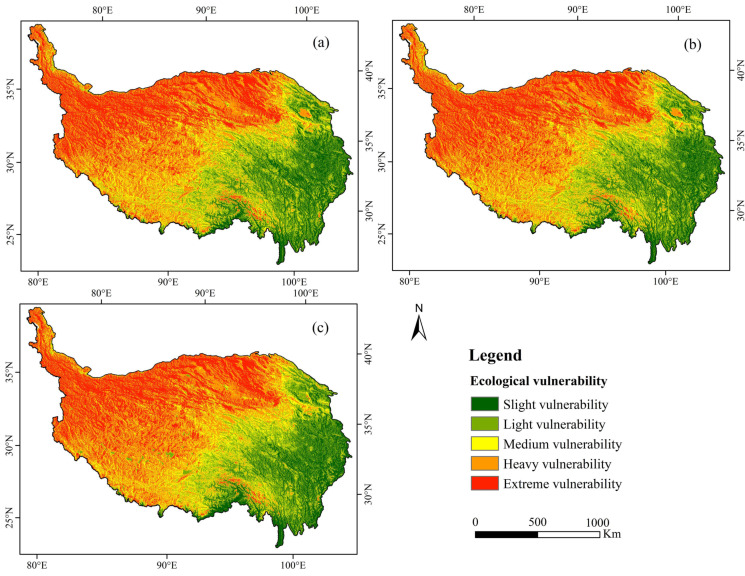
Spatial distribution of ecosystem vulnerability on the Qinghai-Tibet Plateau in (**a**) 2005, (**b**) 2010 and (**c**) 2015.

According to the spatial distribution pattern of EVI classification (Figure 5), the Qinghai-Tibet Plateau as a whole is mainly extremely vulnerable. The overall distribution of ecosystem vulnerability grades was higher in the northwest than in the southeast and gradually increased from southeast to northwest. The ecosystem vulnerability level in the northwest in 2005–2015 was mainly extreme; extremely vulnerable areas first decreased and then increased, whereas for lightly vulnerable areas, the opposite pattern was observed. Ecosystem vulnerability in the southeast was mainly slight and light, with a decrease in slightly vulnerable areas. From southeast to northwest, the vulnerability index increased, and the degree of vulnerability intensified. The middle area mainly showed a medium vulnerability, and the area with medium vulnerability decreased over time.

**Figure 6:**
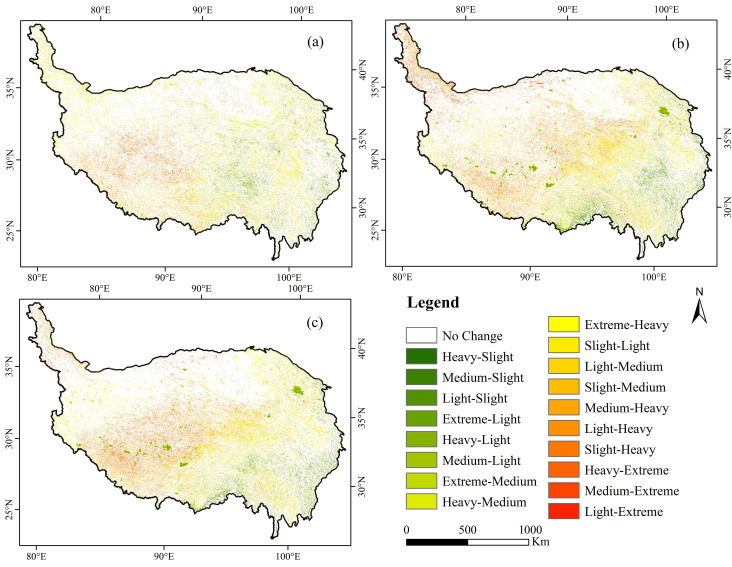
Temporal variations in ecosystem vulnerability on the Qinghai-Tibet Plateau in (**a**) 2005–2010, (**b**) 2010–2015, (**c**) 2005–2010.

To analyze the transition between different levels of ecosystem vulnerability on each patch, we visualized the change in vulnerability grade from 2005 to 2015 (Figure 6). The main changes in ecosystem vulnerability levels consisted of the reduction of slightly and medium vulnerable areas and the increase in heavily and extremely vulnerable areas. From 2005 to 2015, changes in ecosystem vulnerability occurred in 14.80% of the study area, with 18 transformation types. The transition from medium vulnerability to heavy vulnerability accounted for 3.30% of the study area and mainly occurred in the northwest of the Qinghai-Tibet Plateau. The conversion of light vulnerability to medium vulnerability accounted for 2.52% of the study area, mainly in the central region.

### 5.2. Spatial Heterogeneity of Ecosystem Vulnerability

**Figure 7:**
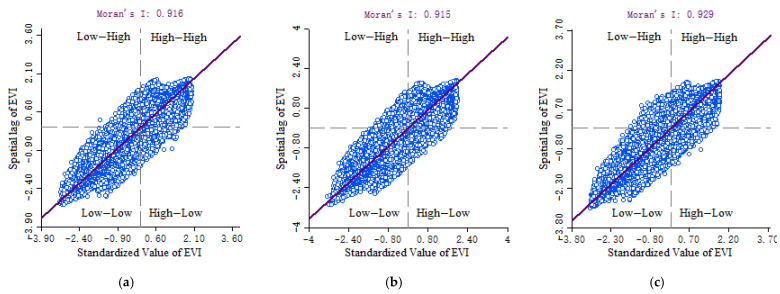
Moran scatterplot of EVI on the Qinghai-Tibet Plateau in (**a**) 2005, (**b**) 2010, (**c**) 2015.

The EVI Global Moran Index for 2005, 2010 and 2015 passed the significance test, with values of 0.916, 0.915 and 0.929 (Figure 7). Ecosystem vulnerability showed positive spatial autocorrelation and high clustering. The overall cluster trend decreased first (from 2005–2010) and then slightly increased (from 2010–2015).

**Figure 8:**
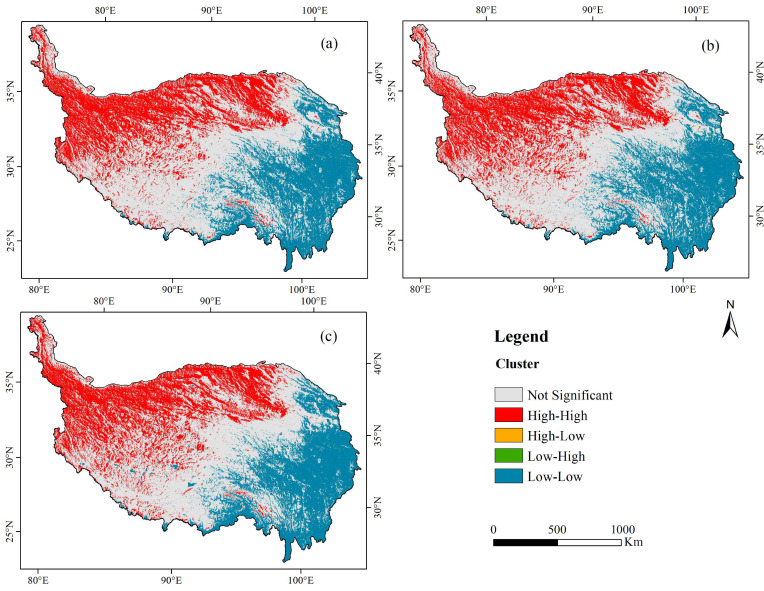
Local spatial autocorrelation diagram for the Qinghai-Tibet Plateau in (**a**) 2005, (**b**) 2010, (**c**) 2015.

Using the local spatial autocorrelation index, the distribution of EVI spatial clustering characteristics and the spatial variation difference on the time scale can be seen intuitively from 2005 to 2015 (Figure 8). In 2005, 2010 and 2015, the distribution of spatial clustering characteristics was similar. The spatial clustering characteristics of EVI on the Qinghai-Tibet Plateau were mainly high-high and low-low. The high-high area was mainly distributed on in the Kunlun Alpine Plateau and in the Qaidam Basin in the northwest of the Qinghai-Tibet Plateau, with heavy and extreme vulnerability. The low-low are was mainly distributed in the southeast, with slight and light vulnerability. The southern part showed insignificant spatial clustering distribution, mainly with medium vulnerability. Compared with other cluster types, the distribution range of the low-high cluster type was lower. The distribution range of the high-low agglomeration was the smallest; such areas were scattered in the transition area from low-low to high-high clusters.

### 5.3. Determinants and Interactions of EVI

In this study, we used the geographic detector method to determine the importance and mutual influence of potential determinants of ecosystem vulnerability. The EVI mean values for the years 2005–2015 were selected as dependent variables, and the corresponding assessment indicators included socio-economic and natural factors. One of the most important findings of this analysis is that natural factors contribute more significantly to EVI than socio-economic factors.

By using factor detectors in geographical detectors, the *q*-statistics of the explanatory power of each influencing factor to ecosystem vulnerability could be obtained (Table 8). The *q*-statistics for natural factors ranged from 0.036 to 0.918, with an average value of 0.449. All factors were statistically significant. The determinants of these factors (in descending order) were the normalized difference vegetation index (NDVI), net primary productivity (NPP), evapotranspiration (ET), annual precipitation (PRE), annual mean temperature (TEM), elevation (ELE), Degree of Relief (DR), slope and surface cutting degree (SCD). These results indicate that vegetation types and climatic characteristics are important determinants of the spatial distribution of ecosystem vulnerability, whereas the effect of topography is relatively weak.

The *q*-statistical values of socio-economic factors ranged between 0.022 and 0.067, with an average value of 0.051 (Table 8). The determinants of the socio-economic factors obtained here can be ranked in descending order of land use ratio (LUR), population density (PD) and GDP density (GDPD). Overall, *q*-statistics show that LUR, PD and GDPD (in descending order) can significantly explain the spatial changes of EVI for the entire Qinghai-Tibet Plateau. The total value ranged between 2.21 and 6.71%.

**Figure 9:**
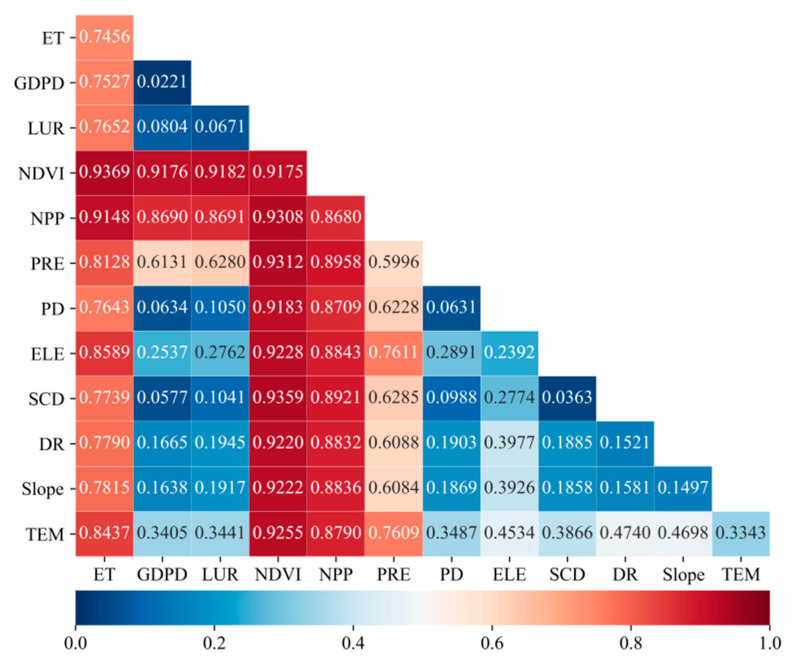
Interactions between pairs of forces influencing EVI. (Notes: the *q*-statistic on the diagonal line in each case denotes the separate effects of each variable (Table 2), whereas the lower periodic matrix includes values for interactive effects between private sources.).

In addition to exploring the effects of single factors on ecosystem vulnerability, we also used the interactive detection module in geographical detectors to analyze the effects of two factors on ecosystem vulnerability. The results show that the interaction between the two factors exceeded that only of a single factor (Figure 9). The effects of NDVI and ET interaction on ecosystem vulnerability were the most significant, indicating that vegetation and surface evapotranspiration were the main factors affecting ecosystem vulnerability on the Qinghai-Tibet Plateau. The *q*-statistics between socio-economic factors were small, but the interaction between the socio-economic and natural factors also strongly affected EVI.

**Figure 10:**
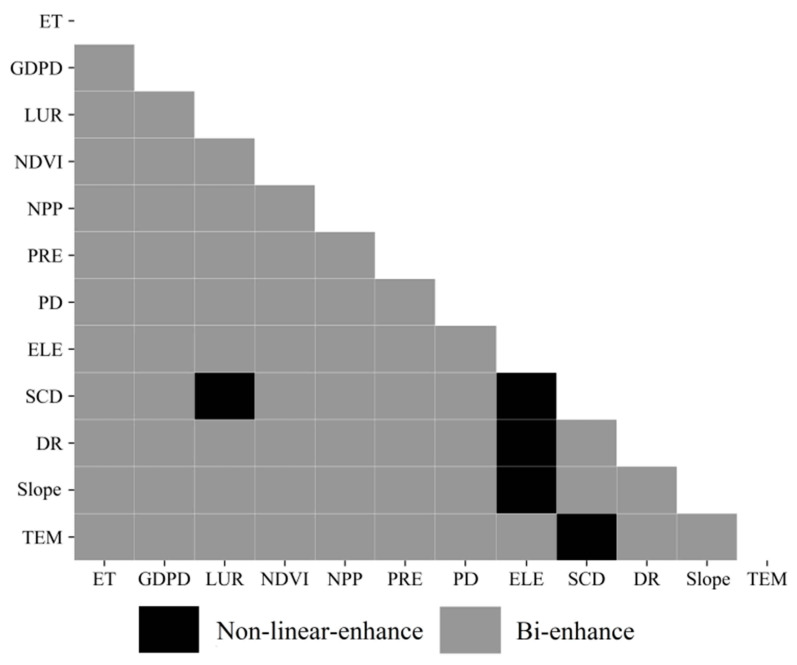
Interaction type between pairs of forces influencing EVI.

There were two types of interactive detection results, namely bi-enhanced and non-linear enhanced effects (Figure 10). Most of the interaction of the two factors showed bi-enhanced effects, and a few showed nonlinear enhanced effects. In fact, the bi-enhanced effects were most often observed for topographic factors (e.g., between elevation and slope and between elevation and slope), which means that the interaction effect was more significant than that produced by a single factor. The interaction effects exhibited nonlinear enhanced effects (such as NDVI, NPP, climate and other factors), indicating that they exceeded the effects of the sum of their individual factors.

## 6. Discussion

### 6.1. Spatial Distribution of Ecosystem Vulnerability

Based on remote sensing data, we used spatial principal components analysis to evaluate the ecosystem vulnerability of the Qinghai-Tibet Plateau for the years 2005, 2010 and 2015 at a spatial resolution of 1 km. The distribution of ecosystem vulnerability showed significant spatial differences, and the overall distribution trend gradually increased from southeast to northwest. The spatial distribution characteristics were similar to those of previous studies on Tibetan Plateau vulnerability [61]. The ecosystem vulnerability of the Qinghai-Tibet Plateau is mainly heavy and extreme, whereas previous studies found medium or heavy vulnerability; these differences might be related to the boundary of the study area and the criteria of vulnerability classification. Previous studies have focused on some areas of the Qinghai-Tibet Plateau, such as the Tibet Plateau [61], the Three-River-Source Area [26], of Delhi City [21]. Compared with previous studies, we expanded the research area to cover the entire Qinghai-Tibet Plateau. However, there is a large desert area in the northwest, resulting in mainly heavy and extreme overall vulnerability.

### 6.2. Effects of Natural and Socio-Economic Factors on Ecological Vulnerability

The *q*-statistical values for natural factors based on GDM ranged from 0.036 to 0.918, with an average of 0.449, whereas those of socio-economic factors were between 0.022 and 0.067, with an average value of 0.051. Therefore, the spatiotemporal variation of EVI mainly depended on natural factors and their changes than on socio-economic factors. Based on analyzing the effects of single factors on vulnerability, we discuss the influences of two factors on vulnerability. The results indicate that NDVI and ET interaction showed the greatest explanatory power to ecosystem vulnerability, instead of NDVI and NPP with the highest single-factor explanatory power. The NPP of the Qinghai-Tibet Plateau decreased gradually from southeast to northwest, showing significant spatial correlation with NDVI, giving it a certain consistency in explaining ecosystem vulnerability. The parameters NDVI and ET can more accurately reflect ecosystem conditions in terms of vegetation and climate than NDVI and NPP interactions. Interaction detection can supplement the analysis results of single-factor detection. A previous study has shown that ecosystem quality is highly positively correlated with NDVI and NPP [26]. Therefore, a decrease in vegetation coverage will inevitably lead to an increase in ecosystem vulnerability. Affected by global warming, rising temperatures with result in increased ET and, subsequently, a loss in soil moisture.

### 6.3. Policy Implications

The Qinghai-Tibet Plateau is an important ecological security barrier for China and even Asia, and the Chinese government attaches great importance to the construction of an ecological civilization on the plateau. Based on the mapping of the vulnerability of the Qinghai-Tibet Plateau ecosystem, the spatial distribution of high- and low-vulnerability areas can be seen. This provides clear evidence for the selection of pilot projects for ecological protection and restoration of the Qinghai-Tibet Plateau. The vegetation types were mainly grassland and desert in the high-vulnerability area in the northwest. Ecological protection and restoration should be therefore be emphasized in this area to prevent grassland degradation and desertification. The vegetation coverage in the low-vulnerability areas in the southeast was high and there were significant human activity impacts. The regional government should therefore improve the ecological compensation mechanism and balance the needs of ecological protection and residential development. In the analysis of factor detection, the *q*-statistics for evapotranspiration and precipitation reached 0.746 and 0.6, respectively, indicating significant effects on ecosystem vulnerability. Therefore, when carrying out ecosystem restoration, it is not only necessary to combine the characteristics of the ecosystem itself, but also to consider the impacts of climate change. For example, Jiang et al. [77] studied the changes in ecosystem services on the Loess Plateau and stated that ecosystem protection needs to consider climate change. In addition, human activities, such as excessive livestock production, which leads to overgrazing, will also have a great impact on the ecological environment. Chen et al. [78], studying the ecosystem of the Mongolian Plateau, showed that the impact of human activities exceeds that of natural environmental changes. Therefore, the future protection of ecosystems should not ignore human interference, and sustainable human activity is a factor to be considered in ecological restoration. For example, a moderate grazing intensity can improve grassland adaptability and reduce grassland vulnerability [27].

### 6.4. Limitations and Future Research Perspectives

In this study, we investigated the influences of natural and socioeconomic factors on the spatial distribution of ecosystem vulnerability. However, there were some limitations and areas of uncertainty. First, ecosystem fragility covers many factors such as nature, economy, society and policies. Due to limitations, such as the inaccessibility of data sources or the difficulty of spatial expression, some indicators compared to other ecosystem vulnerability studies are not included in the indicator system. There is no uniform standard for the selection of sensitivity, resilience and pressure indices. In this paper, climatic conditions are classified as sensitivity index, but some scholars classify them as exposure index (exposure usually refers to the interference degree of environmental and socioeconomic pressure on the ecosystem) [79]. Even if the same ecosystem vulnerability assessment model is selected, climate factors are also divided into different index categories. For example, based on same Exposure-Sensitive-Adaptive model, Jiang et al. [79] takes meteorological factors as exposure, whereas Zheng et al. [80] divides them into sensitivity indicator. Therefore, the scientific index selection method to assess ecosystem vulnerability remains to be explored in depth. Second, the ecosystem vulnerability of the Qinghai-Tibet Plateau was divided into five levels, with only relative differences. For example, the slight vulnerability in this article may be medium or heavy in other areas. Therefore, the classification standard of ecosystem vulnerability is not applicable to areas outside the study area.

## 7. Conclusions

We explored the spatial and temporal differentiation characteristics of the Qinghai-Tibet Plateau ecosystem vulnerability and its driving factors. The Qinghai-Tibet Plateau was mainly in a heavy and extreme vulnerability state from the years 2005 to 2015. The ecosystem vulnerability in the northwest was greater than that in the southeast. The vulnerability grade gradually increased from southeast to northwest. Overall, ecosystem vulnerability deteriorated slightly in 2005–2015. The spatial distribution of EVI showed significant clustering. The high-value area was mainly concentrated in the northwest and the low-values are in the southeast.

The EVI spatial distribution was mainly affected by natural factors. The intensity of these effects followed the order NDVI, NPP, ET, PRE, TEM, ELE, DR, Slope and SCD. Vegetation growth and hydrothermal conditions had significant effects on changes in ecosystem vulnerability. We could also show that socio-economic factors exerted a less significant effect on EVI, on average, than natural factors. The *q*-statistics for these variables followed the order LUR, PD and GDPD. The types of factor interactions were mainly bi-enhanced, with some showing nonlinear enhanced effects. The explanatory power of factor interaction for EVI was greater than that of single factors. The interaction of NDVI and ET had the greatest explanatory power on ecological vulnerability.

Our findings can serve as a scientific base for the establishment of policy implications. Larger efforts are needed to ensure ecological protection and restoration and to prevent grassland degradation and desertification in the high-EVI areas in the northwest. The government should also improve the ecological compensation mechanism and balance ecological protection and residents’ development needs in the southeast. In addition, in the process of ecosystem restoration, it is not only necessary to combine the characteristics of the ecosystem itself, but also to consider the impacts of a changing climate.

## Figures and Tables

**Table 1:** Some definitions of ecosystem vulnerability.

Organization/Author	Definition of Ecosystem Vulnerability
Williams et al. [37]	The potential of an ecosystem to modulate its response to stressors over time and space, where that potential is determined by the characteristics of an ecosystem with many levels of organization. It is an estimate of the inability of an ecosystem to tolerate stressors over time and space.
Birkmann [38]	The sensitive response and self-restoring ability of an ecosystem when it is subjected to external interference. It usually occurs within a specific time and space and is an inherent attribute of the ecosystem.
IPCC [39]	The degree of sensitivity and self-regulation of an ecosystem to disturbances caused by climate change, including extreme weather events.

**Table 2:** Different ecosystem vulnerability assessment indicators.

Year	Study Area	Level Indicators	Secondary Indicators
2017	Yellow River Delta, China [20]	Pressure, support, state, response	Land reclamation rate, population density, human disturbance index, normalized difference vegetation index (NDVI), afforestation area percentage, Shannon’s evenness index, ecological water percentage, pollution load, elastic degree of wetland evaluation; wetland area of change, gross domestic product
2018	Southern Shaanxi, China [10]	Environmental topography and socio-economic level	Cultivation ratio, land use rate, natural growth rate, population density, gross domestic product (GDP) per capita, agricultural output, industrial output, NDVI, average precipitation, average annual temperature, hours of sunshine, average elevation
2018	Jiangsu, China [56]	Pressure, state, response	Soil erosion sensitivity, soil desertification sensitivity, landscape patch density, landscape evenness, land resource use degree
2020	Ningxia Hui Autonomous Region, China [57]	Natural and social factors	Digital elevation model, hours of sunshine, average annual precipitation, average annual temperature, NDVI soil erosion and degree of land use, GDP, agricultural output, industrial output, population density, grassland area
2020	Karst Mountains, China [53]	Sensitivity, resiliency, pressure	Climate, soil, terrain, water, geology, vegetation, land use, social development, economic development

**Table 3:** Basic data and sources of ecological vulnerability assessment for the Qinghai-Tibet Plateau.

Type	Source	Spatial Resolution	Temporal Resolution
NDVI	MODIS/MOD13A3 [59]	1 km	Monthly
Land use	RESDC [60]	1 km	Yearly
DEM	RESDC	1 km	Yearly
Annual average temperature	RESDC	1 km	Yearly
Annual precipitation	RESDC	1 km	Yearly
NPP	MODIS/MOD17A3	1 km	Yearly
ET	MODIS/MOD16A3	500 m	Yearly
Population	RESDC	1 km	Yearly
GDP	RESDC	1 km	Yearly

Notes: NDVI is Normalized difference vegetation index; DEM is digital elevation model; NPP is Net Primary Productivity; ET is Evapotranspiration; GDP is gross domestic product; RESDC is Resource and Environment Science and Data Center, Chinese Academy of Sciences.

**Table 4:** Ecosystem vulnerability assessment indicators for the Qinghai-Tibet Plateau.

Factor Category	Indicator	Type
Sensitivity	Annual precipitation (PRE)	−
	Annual average temperature (TEM)	−
	Evapotranspiration (ET)	−
	Elevation (ELE)	+
	Slope	+
	Surface cutting depth (SCD)	+
	Degree of relief (DR)	+
Resilience	Normalized difference vegetation index (NDVI)	−
	Net Primary Productivity (NPP)	−
Pressure	Population density (PD)	+
	Gross domestic product density (GDPD)	+
	Land use rate (LUR)	+

Note: “+” means positive action; the greater the value, the lower the quality of the ecological environment, the greater the probability of a fragile ecological environment; ”−” means reverse action.

**Table 5:** Results of the SPCA (spatial principal components analysis) of ecosystem vulnerability on the Qinghai-Tibet Plateau.

PC	Eigenvalues			Contribution Ratio of Eigenvalues/%			Cumulative Contribution of Eigenvalues/%		
	2005	2010	2015	2005	2010	2015	2005	2010	2015
1	0.0669	0.0729	0.0763	48.7327	48.5814	51.9332	48.7327	48.5814	51.9332
2	0.0391	0.0429	0.0392	28.5202	28.5649	26.6587	77.2529	77.1463	78.5920
3	0.0101	0.0105	0.0092	7.3796	7.0087	6.2786	84.6325	84.1550	84.8706
4	0.0070	0.0079	0.0072	5.0880	5.2544	4.8803	89.7204	89.4094	89.7509

**Table 6:** Classification of the ecosystem vulnerability index (EVI).

EVI	Slight	Light	Medium	Heavy	Extreme
Grading standard	<0.35	0.35–0.5	0.5–0.64	0.64–0.77	>0.77

**Table 7:** Interaction Detector Model.

Description	Interaction Type
*q* (X1∩X2) < Min (*q* (X1), *q* (X2))	Non-linear-weaken
Min(*q* (X1), *q* (X2)) < *q* (X1∩X2) < Max(*q* (X1)), *q* (X2))	Uni-weaken
*q* (X1∩X2) > Max (*q* (X1), *q* (X2))	Bi-enhance
*q* (X1∩X2) = *q* (X1) + *q* (X2)	Independent
*q* (X1∩X2) > *q* (X1) + *q* (X2)	Non-linear-enhance

Note: *q* (X1∩X2) represents the interaction effect of influencing factors X1 and X2, and *q* (X1) and *q* (X2) represent the respective effects of X1 and X2, respectively.

**Table 8:** Results for different factors of EVI.

Factors	NDVI	NPP	ET	PRE	TEM	ELE	DR	Slope	LUR	PD	SCD	GDPD
*q* statistic	0.918	0.868	0.746	0.600	0.334	0.239	0.152	0.150	0.067	0.063	0.036	0.022
*p* Value	0.000	0.000	0.000	0.000	0.000	0.000	0.000	0.000	0.000	0.000	0.000	0.000

## Data Availability

All relevant data sets in this study are described in the manuscript.
